# Reformulation of Emulsion-Type Pork Sausage Using Collagen and Plasma Proteins as Soy Protein Substitutes for Soy-Free Product Development

**DOI:** 10.3390/gels12060545

**Published:** 2026-06-18

**Authors:** Ionela Ramona Gheorghe (Pîrvu), Violeta Nour, Georgiana Gabriela Codină

**Affiliations:** 1 Faculty of Food Science and Engineering, “Dunărea de Jos” University of Galati, Domnească Street 111, 800201 Galati, Romania; ramona.pirvu@teldo.ro; 2 Department of Horticulture & Food Science, University of Craiova, 13 AI Cuza Street, 200585 Craiova, Romania; 3 Faculty of Food Engineering, Stefan cel Mare University of Suceava, 720229 Suceava, Romania; codina@fia.usv.ro

**Keywords:** soy protein, collagen, plasma protein, carrageenan, xanthan gum, polyphosphates, techno-functional properties

## Abstract

The present study evaluated the feasibility of replacing soy protein isolate with collagen and plasma proteins, either individually or in combination with κ-carrageenan, xanthan gum, and sodium tripolyphosphate, in an emulsion-type pork sausage, based on selected physicochemical, compositional, and textural quality parameters. Six formulations were produced, including a control and five reformulated variants in which soy protein was fully replaced by a mixture of collagen (1.88%) and plasma proteins (3.4%), used alone or supplemented with κ-carrageenan (1.0%), xanthan gum (0.2%), and sodium tripolyphosphate (0.2%). Moisture, protein, fat and collagen contents, color, pH, and sensory properties were analyzed after processing, while TBARS values and textural properties were assessed initially and after 30 days of storage. As a result of the reformulation, collagen content increased by 32.35–40.33%, while the collagen-to-protein ratio remained within legal limits (<20%). Soy protein replacement increased textural parameters, including hardness, cohesiveness, gumminess, chewiness, and shear force. Carrageenan and sodium tripolyphosphate enhanced texture and oxidative stability, whereas xanthan gum negatively affected texture quality and sensory acceptance. The formulation containing collagen, plasma proteins, carrageenan (1%) and sodium tripolyphosphate (0.2%) achieved the highest sensory scores, comparable to those of the control. The results show that replacing soy protein in an emulsion-type pork sausage is feasible when using optimized combinations of collagen, plasma proteins, and κ-carrageenan systems.

## 1. Introduction

Meat products play a significant role in many people’s diets, largely because of their appealing taste and relatively low cost. They are complex food systems where functional properties such as water-binding capacity, gelation behavior, and emulsifying ability play key roles in determining their stability and texture [1]. The quality attributes of meat products, including appearance, texture, and palatability, are largely determined by the functional properties of their proteins. In meat products, myofibrillar proteins primarily contribute through key techno-functional properties such as gel formation, particle binding and adhesion, emulsification, and water-holding capacity. However, meat proteins typically exhibit limited techno-functional performance, often necessitating the addition of other ingredients to achieve a stable emulsion system. High meat prices have prompted the food industry to incorporate non-meat protein ingredients into meat products, both as a cost-effective source of high-quality protein and as functional enhancers. These additives help improve textural attributes such as adhesion and firmness, enhance emulsion stability, minimize losses during heat processing, increase product yield, and ultimately lower production costs [2].

Soy provides a high-quality protein source, owing to its well-balanced amino acid profile. Beyond its nutritional value, soy protein exhibits important functional properties in food systems. Consequently, the incorporation of soy—particularly as protein isolates and concentrates—has become highly significant in the food industry, offering promising possibilities for developing meat products that are both healthier and more sustainable [3]. Soy protein is widely used in meat products as a binder to improve yields, as a gelling agent to enhance emulsion stability, and as a meat substitute to reduce costs [4]. Soy protein increases fat absorption, water binding capacity and adhesion of minced meat [5]. The amount of soy protein isolate incorporated varies between 1% and 5%, depending on both the specific protein ingredient and the type of meat product being formulated. Many emulsified meat formulations containing soy protein products have excellent visual appearance, good texture, no off-flavors, and have reduced cooking losses and higher yields while maintaining good nutritional quality. Soy protein isolates and concentrates are the most effective soy ingredients used in meat preparations [6]. However, soy protein is recognized as a major food allergen [7,8] and is included in the World Health Organization’s list of the “big 8” allergens, which collectively account for approximately 90% of all reported food allergy cases in the United States [9]. The allergenicity of soy protein is mainly associated with the seed storage proteins Gly m 5 (β-conglycinin) and Gly m 6 (glycinin), which possess multiple IgE-binding epitopes and show considerable stability to heat treatment and enzymatic digestion, characteristics that contribute to the persistence of allergic reactions even after food processing [8,10]. Unfortunately, the prevalence of soy allergies has been increasing, with a growing number of individuals affected over time [11,12]. The use of soy protein in meat products is regulated and restricted in several countries. For example, in Brazil, processed meat products may contain up to 4% soy protein, whereas in the United States the permitted level can reach as high as 12%. For the industrial sector, this addition is very important, as it helps lower production costs while enhancing technological performance. It can also slow lipid oxidation and function as a partial replacement for both meat and fat components [13]. Due to the allergenicity of soy proteins, food manufacturers are forced to look for alternative protein sources [14,15]. At the same time, the meat industry produces by-products such as blood and collagen, which can be processed into high-value ingredients with strong techno-functional potential, including plasma proteins and collagen-based proteins [3,16]. More efficient utilization of meat by-products is significant not only for economic reasons but also in terms of environmental sustainability. Recent studies have demonstrated that collagen hydrolysates can improve water-holding capacity, emulsion stability, texture, oxidative stability, and nutritional quality of sausages [17,18], whereas plasma proteins contribute primarily through their excellent water-binding, emulsifying, and heat-induced gel-forming properties, resulting in enhanced texture, processing yield, and stability of emulsified meat products [19,20,21]. Plasma proteins have the potential to structure meat systems and modulate their texture; however, their application as techno-functional ingredients in meat formulations remains relatively limited at present [22,23].

Previous studies have demonstrated that collagen and plasma proteins can partially or fully replace plant-based binders in processed meat products without compromising technological quality [19,24]. Nevertheless, limited research has evaluated their combined application as a direct substitute for soy protein in emulsion-type sausages, particularly with regard to maintaining product functionality while reducing allergenic risk.

To mimic the techno-functional properties of soy protein, collagen and plasma proteins can be used in combination with polysaccharides. Alginate, inulin, carrageenan, konjac, xanthan, and chitosan have been previously applied in emulsified gel systems as gelling agents, owing to their hydrophilic nature, high molecular weight, and gel-forming ability [25,26]. Carrageenan, a sulfated anionic polysaccharide derived from marine red algae, is employed in the food industry as a stabilizing, gelling and thickening agent, as well as a fat substitute [27]. Xanthan is a polysaccharide used in the food industry for its ability to retain water and enhance texture, and it is primarily used as a stabilizer and thickening agent in emulsions, as well as a gelling agent in various food systems [28]. Several studies have investigated the application of carrageenan and xanthan in meat products [29,30,31]. Their findings indicated that incorporation of these hydrocolloids notably decreased cooking losses and improved emulsion stability. For example, Cao et al. [30] demonstrated that the addition of κ-carrageenan at 0.2% (*w*/*w*) significantly improved the textural and gel properties of frankfurters, as well as the rheological behavior of the meat batter. However, to the best of our knowledge, only a limited number of studies have examined their combined use with collagen and plasma proteins. Zhao et al. [23] investigated the effect of porcine plasma protein and xanthan gum-based systems on the quality characteristics of low-fat sausages.

The present study aimed to fully replace soy protein as a techno-functional ingredient in an emulsion-type pork sausage with a combination of collagen and plasma proteins, in order to produce a soy-free product while maintaining acceptable textural and sensory quality. To achieve this objective, the effects of replacing soy protein with a mixture of collagen and plasma proteins, alone or in combination with carrageenan, xanthan gum, and sodium tripolyphosphate, on the physicochemical, textural, and sensory properties of sausages were evaluated.

## 2. Results and Discussion

### 2.1. Compositional Characteristics

Collagen and plasma proteins, derived from by-products of the meat industry, exhibit strong potential for high-value techno-functional applications. However, their utilization as meat extenders in meat products remains limited [3]. The compositional characteristics of control and reformulated sausage samples are presented in Table 1.

The moisture content ranged from 58.06% to 61.30%, protein content from 16.52% to 17.20%, fat content from 17.53% to 22.09% and collagen content from 2.38% to 3.34%. All formulations were in compliance with the Romanian technical regulation governing emulsion-type sausage quality [32], which sets maximum limits of 66% for moisture and 30% for fat, as well as a minimum protein content of 15% in the final product. Regarding the collagen content, the collagen-to-protein ratio remained below 20%, complying with the maximum limit stipulated by Romanian legislation [32] for this product. No statistically significant differences (*p* < 0.05) were detected in total protein content among the samples. Consequently, replacing soy protein ingredients with collagen and plasma proteins minimized the allergenic potential associated with soy, while preserving the overall protein content of the final product. However, substituting soy protein with collagen and plasma protein resulted in a significant increase in collagen content (by 32.35–40.33%), along with an elevated collagen-to-protein ratio across all reformulated variants.

Replacing soy protein with collagen and plasma proteins (without other additives) led to a significant reduction in lipid content, likely due to the lower intrinsic fat content and reduced fat-binding capacity of collagen + plasma protein ingredients compared with soy protein (reduction of 10.46%). Furthermore, the incorporation of carrageenan increased the fat content of the sausage samples, and a similar effect was observed with xanthan gum, suggesting an enhanced fat retention capacity conferred by these hydrocolloids. κ-Carrageenan is a functional hydrocolloid used in emulsified meat products to improve structural stability as well as water and fat retention through gel network formation [33,34]. Similarly, xanthan gum has been reported to enhance product stability and fat-binding properties in meat systems [23,35,36]. The addition of polyphosphates also improved fat retention in the formulations, which can be attributed to their polyelectrolyte nature that increases ionic strength, promotes myofibrillar protein solubilization, and thereby enhances water-holding and fat-emulsifying capacity [37,38].

The moisture content slightly increased after total replacement of soy protein with collagen + plasma proteins, without significant differences, demonstrating a good water retention capacity of these proteins. In a previous study, Álvarez et al. [16], investigating the substitution of pork meat with protein recovered from meat co-products in Irish breakfast sausage formulations, reported a strong water-holding capacity of plasma proteins. Collagen hydrolysates may act as emulsifying agents in emulsion-based systems and function as fat replacers in low-fat meat products [39]. Hydrolyzed collagen has been incorporated in processed meat products, such as sausages, where it has been used to replace pork fat at a substitution level of up to 50%. Sousa et al. [40] highlighted the effectiveness of hydrolyzed collagen in retaining water in Frankfurter-type sausages. Lee and Chin [41] reported that low-fat sausages formulated with added pork gelatin exhibited good water-holding capacity, which contributed to reduced cooking losses. Choe and Kim [42] also reported that the incorporation of collagen-based mixtures containing konjac, carrageenan, or tapioca starch significantly increased the moisture content of marinated pork loin ham samples, an effect partly attributed to the ability of collagen to immobilize water during cooking. Plasma proteins have also been evaluated as fat replacers in meat products, yielding favorable results, especially in combination with hydrocolloids [25]. The addition of carrageenan, xanthan gum, and sodium tripolyphosphate did not significantly affect (*p* < 0.05) the protein and collagen contents of the reformulated products.

### 2.2. Color Properties and pH

The results on the instrumental color analysis of the sausage formulations are shown in Table 2. The substitution of soy protein with collagen protein led to a significant increase in L* values, indicating a lighter appearance. Pereira et al. [43] also reported that the addition of collagen fibers contributed to increased lightness in frankfurter-type sausages. No significant differences were observed in a* values among these samples. The incorporation of non-meat ingredients into meat products may result in increased lightness values [44]. The addition of hydrocolloids (carrageenan or xanthan) resulted in a slight reduction in L* and a* values, indicating a lighter and less intense red coloration, likely due to dilution of the myoglobin pigment [13]. Furthermore, the addition of sodium tripolyphosphate increased the a* values to levels that did not differ significantly from those of the control sample. Demirci et al. [45] also reported that increasing the addition of gums (carrageenan or xanthan) led to a decrease in redness in both raw and cooked meatball samples.

Sausage samples reformulated by replacing soy protein with collagen and plasma proteins exhibited higher yellowness (b*) values compared with the control. Similarly, Sousa et al. [40] reported increased b* values following partial fat replacement with hydrolyzed collagen, attributing this effect to the higher intrinsic yellow coloration of hydrolyzed collagen compared with pork fat. Prabhu et al. [46] also reported that the addition of pork collagen significantly (*p* < 0.05) increased internal b* values in frankfurter formulations at inclusion levels of 2% and above. In contrast, Bertolo et al. [1] observed no significant differences (*p* > 0.05) in either raw or cooked b* values in burgers following the replacement of textured soy protein with brewing yeast cells. Similarly, Pereira et al. [43] reported that the addition of collagen fiber in frankfurter-type sausages had no significant effect on yellowness (b*) values.

The replacement of soy protein with collagen and plasma proteins resulted in a significant increase (*p* < 0.05) in hue angle, indicating a shift from red toward yellowish tones and reflecting a reduction in redness and a less desirable meat color. However, the addition of hydrocolloids (carrageenan or xanthan) reduced the hue angle to values that did not differ significantly (*p* > 0.05) from the control sample formulated with soy protein. The addition of sodium tripolyphosphate to the reformulated samples, made with collagen, plasma proteins, and hydrocolloids (carrageenan or xanthan), resulted in a further slight increase in hue angle, indicating an additional shift toward less red and more yellowish tones and a slight deterioration in color quality. The observed increase in hue angle following the addition of sodium tripolyphosphate in combination with hydrocolloids may be attributed to synergistic modifications in protein functionality and microstructural organization. Tripolyphosphate increases pH and ionic strength, promoting myofibrillar protein swelling, solubilization, and improved water-holding capacity through enhanced electrostatic repulsion and protein unfolding. Concurrently, hydrocolloids such as carrageenan and xanthan form highly hydrated networks that immobilize water and increase system viscosity, thereby altering the spatial distribution of aqueous and lipid phases. These structural changes enhance light scattering within the matrix and may dilute the visual intensity of myoglobin-derived red pigments. In addition, phosphate-induced changes in protein–pigment interactions may influence the chemical state of myoglobin, further contributing to reduced redness. The ΔE value of the PC sample was greater than 4, indicating a clearly perceivable color difference between the control sample formulated with soy protein and the reformulated sample in which soy protein was replaced by a collagen–plasma protein mixture. In contrast, ΔE values for the other reformulated samples ranged between 2.0 and 3.5, suggesting that any color differences relative to the control were only moderately perceptible and typically discernible upon close visual inspection.

The control emulsion-type sausage formulated with soy protein exhibited the highest pH value. The replacement of soy protein with collagen and plasma proteins resulted in a significant decrease (*p* < 0.05) in pH; however, the addition of carrageenan restored the pH to levels close to those of the soy-based control. Previous studies have also noticed that changes in pH depend on the intrinsic pH of the added non-meat ingredient as well as its interactions with the meat matrix [2].

### 2.3. Lipid Oxidation

**Figure 1:**
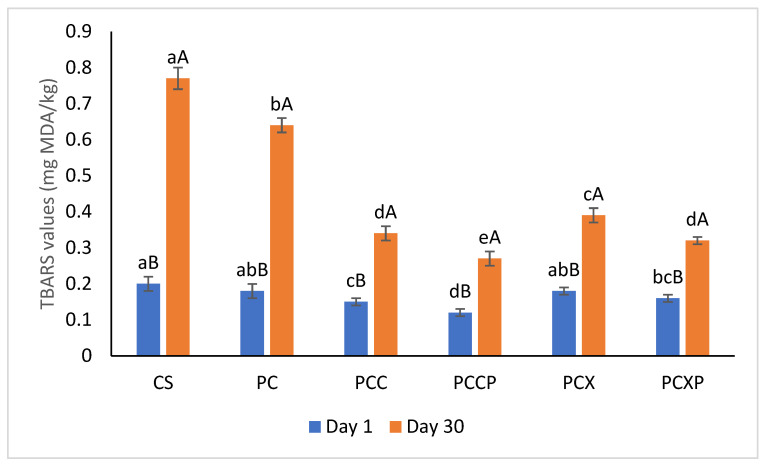
TBARS values (mg MDA/kg) of sausage samples; different lowercase letters on the bar indicate significant differences (*p* < 0.05) between samples for the same storage period while different uppercase letters are indicative of significant differences between sampling times for the same sausage formulation (*p* < 0.05); CS—sausage made with soy gel and soy protein isolate (control); PC—sausage reformulated by replacing soy protein with collagen + plasma proteins; PCC—sausage reformulated by replacing soy protein with collagen + plasma proteins + 1% carrageenan; PCCP—sausage reformulated by replacing soy protein with collagen + plasma proteins + 1% carrageenan + 0.2% sodium tripolyphosphate; PCX—sausage reformulated by replacing soy protein with collagen + plasma proteins + 0.2% xanthan gum; PCXP—sausage reformulated by replacing soy protein with collagen + plasma proteins + 0.2% xanthan gum + 0.2% sodium tripolyphosphate.

The results of the TBARS values in control and reformulated sausage samples at 0 and 30 days of refrigerated storage (4 °C) are presented in Figure 1. TBARS value reflects the concentration of compounds capable of reacting with thiobarbituric acid in meat products, predominantly malondialdehyde, a secondary product of lipid oxidation, serving as a direct indicator of the extent of oxidative deterioration in these products. Lower TBARS values are commonly linked to enhanced flavor stability and a lower formation of rancid off-flavors during refrigerated storage [47].

As illustrated in Figure 1, TBARS values exhibited an overall increasing trend throughout the sausage storage period. However, after 30 days of storage, all formulations exhibited TBARS values below the 1.0 mg MDA/kg threshold associated with the perceptible onset of rancidity [47]. TBARS values in reformulated samples were slightly reduced in the first day after processing as compared with the control. The TBARS ranged from 0.20 to 0.77 and 0.18 to 0.64 mg/kg in the CS and PC samples, respectively, during the 30 days of refrigerated storage, indicating that replacing soy proteins with collagen + plasma proteins could slightly inhibit the increase in TBARS value in sausage. These results might be explained by the antioxidant activity of collagen and plasma proteins that prevented lipid oxidation [48]. Previous results indicated the presence in collagen hydrolysate of bioactive amino acids possessing antioxidant activity, including arginine, histidine, and methionine [42,49]. Moreover, the bioactive peptides derived from collagen hydrolysates may exert electron-donating and metal-chelating activities, thereby reducing both lipid and protein oxidation pathways [50,51]. In addition, some previous studies have shown that different hydrolysates of plasma from deer, sheep and pork using plant and fungal proteases showed antioxidant properties [51,52,53].

Supplementation with carrageenan and xanthan further inhibited oxidative processes during storage, as evidenced by significantly lower (*p* < 0.05) TBARS values in PCC and PCX samples compared to the PC sample. Previous studies revealed that lambda- and k-carrageenan exhibited significant antioxidant activity [54,55]. In turn, xanthan gum demonstrated notable antioxidant potential, as evidenced by its capacity to scavenge free radicals and effectively suppress lipid peroxidation processes [56,57].

Sausage samples supplemented with polyphosphates exhibited significantly smaller changes in TBARS values over 30 days of storage compared to their counterparts without polyphosphate addition. Phosphates are extensively incorporated into processed meat products due to their versatile technological functionalities, among which their contribution to enhancing oxidative stability is particularly noteworthy [58]. Furthermore, phosphates exhibit strong antioxidant activity in muscle foods by chelating pro-oxidant metal ions that catalyze lipid oxidation; once bound, these ions remain present but are no longer able to participate in oxidative reactions [59].

### 2.4. Textural Properties

In meat processing, soy protein is the predominant plant-derived ingredient, valued primarily for its emulsifying and stabilizing functionality, as well as for its capacity to enhance water-holding capacity and improve product texture [1]. Consequently, its substitution presents significant technological challenges, particularly in maintaining the desired textural properties of meat products. The mean values of the textural parameters for both control and reformulated sausage samples are shown in Table 3. Substituting soy protein with a combination of collagen and plasma proteins resulted in significant increases in hardness, adhesiveness, cohesiveness, resilience, gumminess, chewiness, and shear force of the sausage samples. The observed increase in hardness may be associated with enhanced water retention within the protein matrix, which promotes a more compact and structured gel network, thereby improving texture, cohesion, and firmness of the final product [43]. Consistent with the present findings, Ham et al. [18] also reported that the incorporation of 1% duck skin gelatin hydrolysate enhanced the cohesiveness and chewiness of cooked sausages, while Sousa et al. [40] found that partial replacement of pork backfat with collagen hydrolysate in frankfurter-type sausages resulted in increased hardness, elasticity, and chewiness. Regarding plasma protein addition, Álvarez et al. [16] previously reported that substituting 10% of pork meat with blood plasma in Irish breakfast sausages increased hardness, chewiness, gumminess, and cohesiveness, whereas at a 20% replacement level, no significant differences in these textural attributes were observed compared with the control sample. The replacement of 10% pork meat with plasma was also accompanied by significant increases in water-holding capacity [16].

The incorporation of carrageenan into the formulation based on collagen and plasma protein led to additional increases in hardness, cohesiveness, and shear force, while simultaneously decreasing adhesiveness. Çelebi & Erge [34] found also that incorporation of carrageenan at 1% level increased the hardness of Bologna-type chicken sausages while Candogan and Kolsarici [60] demonstrated that the addition of carrageenan to low-fat frankfurters improved emulsion stability, with concentrations below 0.5% enhancing gel elasticity and higher levels (0.5–1.5%) increasing hardness. Carrageenans have been widely incorporated into comminuted meat products to enhance structural stability and textural properties, particularly in low-fat formulations [60]. Moreover, their addition has been shown to significantly increase hardness, chewiness, and gumminess [45,61]. The effect of carrageenan on water retention capacity, hardness, and elasticity is attributed to its distribution within the protein gel matrix: at low concentrations, it is located within the interstitial spaces of the network, whereas at higher concentrations, it contributes to the formation of an additional carrageenan gel network [62,63].

However, the incorporation of xanthan gum led to a significant reduction in hardness, shear force, cohesiveness, gumminess, and chewiness, both compared to the soy protein-based control and to the sample reformulated with collagen and plasma proteins. Majzoobi et al. [64] also observed that κ-carrageenan increased hardness and chewiness, whereas xanthan gum—especially at levels above 0.6%—decreased hardness, springiness, cohesiveness, and chewiness in meat-free sausages. Xanthan gum is a long chain polysaccharide composed of glucose, mannose, and glucuronic acid that is characterized by its ability to substantially increase the viscosity of liquids at very low concentrations. A previous study reported that the incorporation of 0.5% xanthan gum into low-fat breakfast sausages improved fat and moisture retention, although it adversely affected textural and sensory properties [65]. The negative effect of xanthan gum on texture quality may be attributed to its strong water-binding capacity and its interference with protein network formation. At higher concentrations, xanthan gum can compete with meat proteins for available water, resulting in a weaker gel matrix, reduced firmness and elasticity, and a gummy mouthfeel. Similar observations were reported by Solheim and Ellekjær [66], who found xanthan-containing sausages to be less firm and less elastic, and by Wang et al. [67], who showed that xanthan gum promoted the formation of weaker and more disintegrated gel structures in emulsified sausages.

The incorporation of polyphosphates did not exert a significant effect on hardness; however, it led to a reduction in cohesiveness, gumminess, and chewiness. Phosphates are widely used in processed meats for their ability to improve water holding capacity, myofibrillar protein solubilization, emulsion stability and cooking yield [58]. Gadekar et al. [68] previously reported that addition of phosphate increased water holding capacity and cooking yield in goat meat sausages, while hardness and chewiness decreased. Other studies highlighted the ability of phosphates to improve tenderness in ground meat [69].

After 30 days of refrigerated storage, hardness, adhesiveness, cohesiveness, gumminess, and chewiness increased in both control and reformulated samples, except for those containing xanthan gum, which showed a slight decreasing trend. Previous studies have reported similar trends in textural properties of various emulsion-type sausage products and attributed these changes to the loss of moisture and water migration within the bars during storage [70,71].

### 2.5. Sensory Evaluation

The sensory scores for the attributes evaluated are presented in Table 4. Significant differences were found between sausage formulations. Except color, the control sausage exhibited the highest scores for all sensory attributes. The reformulation by replacing soy protein with collagen and plasma proteins determined significant decreases (*p* < 0.05) in all sensory attributes. The most prominent differences were observed for the appearance, taste and texture. The incorporation of carrageenan, either alone or in combination with polyphosphates, led to increased scores in reformulated sausage made by replacing soy protein with collagen and plasma proteins. In a previous study, sensory evaluation of cooked turkey meat sausages indicated that carrageenan addition did not significantly affect taste (*p* > 0.05), except for a slight decrease at 1.5%, while progressively enhancing texture and appearance acceptability with increasing concentrations—showing no significant differences at 0.2%, a significant improvement at 0.5% (*p* < 0.05), and highest overall panelist acceptance at 0.8% [62]. In contrast, Çelebi & Erge [34] reported that carrageenan incorporation in chicken sausages did not affect color or tenderness, but significantly reduced juiciness and overall acceptability (*p* < 0.05).

No significant differences (*p* > 0.05) were observed for appearance, color, flavor and general acceptability between reformulated sausage made with 1% carrageenan and 0.2% sodium tripolyphosphate addition (PCCP) and control. PCCP slightly surpassed the control in terms of color, possibly due to enhanced pigment stability associated with the presence of polyphosphates and the animal protein matrix. Carrageenan exhibited good functional compatibility with animal proteins (collagen and plasma proteins) while polyphosphates added in a low dose (0.2%) significantly contributed to optimizing texture and sensory perception. The results of the LSD test (Table 4) indicated that sausage samples reformulated with collagen and plasma proteins and supplemented with carrageenan and sodium tripolyphosphate exhibited comparable consumer preference, with no significant differences observed relative to the control sausage made with soy protein. The instrumental texture analysis indicated significant increases in hardness, chewiness, and related textural parameters in these reformulated sausage samples. Despite the observed increases in instrumental firmness, the sensory evaluation did not reveal a decrease in overall acceptability. These formulations remained within the acceptable range for emulsified-type sausages, suggesting that the textural modifications were not perceived as detrimental by the trained sensory panel under the conditions of this study. It should be noted, however, that instrumental texture parameters do not always directly correlate with consumer perception, particularly in emulsified meat products where moderate firmness may be desirable to ensure product integrity. Therefore, while the present results indicate acceptable sensory performance, broader consumer studies would be required to confirm market acceptance.

Xanthan gum, at the concentration employed (0.20%), appears to have adversely affected textural properties, flavor, taste and overall acceptability. As a result, the formulations containing xanthan showed scores in the range of the rejection threshold. These results may be attributed to the batter’s inability to form a stable gel, along with the slimy mouthfeel and distinctive taste associated with xanthan. Xanthan gum tends to increase viscosity rather than build a strong gel network. As a result, it may disrupt the meat protein structure, leading to softer texture, lower cohesiveness, and undesirable mouthfeel, along with diminished sensory acceptance [67]. Xanthan gum is often associated with a slick or mucilaginous sensation, especially at higher levels. It can entrap flavor compounds or slow their release, resulting in muted flavor perception and less intense meat taste. Such characteristics are generally perceived as undesirable in meat products, where consumers expect a firm, elastic, and fibrous texture rather than a gel-like consistency.

Demirci et al. [45] found that increase in xanthan, guar and carrageenan gum addition levels significantly decreased (*p* < 0.05) color, taste, hardness and overall palatability of meatballs. In contrast, Majzoobi et al. [64] showed that addition of 0.3% and 0.6% κ-carrageenan significantly (*p* < 0.05) improved the general acceptability of the meat-free sausages while inclusion of xanthan gum made no significant difference (*p* > 0.05) in the overall acceptability of the samples compared to the control. A greater juiciness, associated with a strong water retention capacity, has been reported in several previous studies as a result of incorporating xanthan into meat products [23,29]. However, Cîrstea et al. [29] reported that the use of xanthan as cold gelling agent led to a crumbly structure in burgers, negatively impacting their overall acceptability.

## 3. Conclusions

Soy protein exhibits highly valuable techno-functional properties in meat products; however, its high allergenic potential compels meat processors to seek alternative ingredients in order to maintain product quality and economic efficiency. The aim of the present study was to evaluate the effects of replacing soy protein with a mixture of collagen and plasma proteins, either alone or in combination with carrageenan, xanthan gum, and sodium tripolyphosphate, on the physicochemical, textural, and sensory characteristics of emulsion-type pork sausage. The reformulation did not change the total protein and fat contents, whereas collagen content increased, although the collagen-to-protein ratio remained within the legal limits. Substituting soy protein with collagen + plasma proteins resulted in significant increases in hardness, adhesiveness, cohesiveness, gumminess, chewiness, and shear-force of the sausage samples. In terms of textural parameters, addition of carrageenan increased hardness, cohesiveness, and shear force while xanthan reduced texture quality. Reformulated sausage with carrageenan (1%) and sodium tripolyphosphate (0.2%) addition achieved the highest sensory and overall acceptability scores, with no significant differences from the control. The results demonstrated that soy protein replacement in emulsion-type sausage is technologically feasible when optimized combinations of collagen, plasma proteins, and κ-carrageenan-based systems are used, while xanthan gum requires careful formulation control. Future studies should focus on the nutritional and sensory optimization of emulsion-type sausage formulations made without soy protein as a techno-functional ingredient.

## 4. Materials and Methods

### 4.1. Materials

Pig carcasses were purchased postmortem from Palaloga Carnprep Resita S.R.L. (Bocșa, Romania), a licensed abattoir. Combi Gel P, containing pig plasma protein, and Combi Gel PP containing pig collagen protein, were purchased from Aromatique Food S.R.L. (Bucharest, Romania) while soy protein isolate Izosoy 90 (90% protein concentration) was procured from S.C. Dar International S.R.L. (Bucharest, Romania). Sodium lactate (Bactostop Fresh Liquid), additives and spices mixture (Blend Salam SDW 2), sodium tripolyphosphate, carrageenan and xanthan gum were purchased from S.C. Aromatique Food S.R.L. (Bucharest, Romania) while nitrite curing salt (containing 0.4–0.6% sodium nitrite) was from Crinexcom S.R.L. (Galati, Romania).

### 4.2. Preparation of Emulsion-Type Sausage Samples

A total of six emulsion-type sausage formulations were produced, and the composition of each batter is presented in Table 5. One formulation served as the control (CS) and contained soy gel and soy protein isolate. The remaining five formulations were produced by fully replacing the soy-based ingredients with collagen and plasma proteins. Among these, one contained no additional additives (PC), two were supplemented with carrageenan (PCC and PCCP), and two included xanthan gum (PCX and PCXP). Sodium tripolyphosphate was also incorporated into the PCCP and PCXP variants.

The “Sandwich” emulsion-type sausage was produced at an industrial scale from pork trimmings, leg meat, and backfat. After grinding in a mincer Kolbe Foodtec (MultiGrind AW, Elchingen, Germany), and homogenizing the ingredients in a mixer Inotec/Handtmann model IM-500 (Inotec, Biberach an der Riß, Germany), according to the formulations in Table 5, the batter was stuffed into rehydrated ø 90 mm fiber membrane using a sausage filler Handtmann model VF 616 (Handtmann, Biberach an der Riß, Germany). The stuffed sausage casings were linked to a length of 15 cm each (about 0.5 kg). After linking, all six sausage batches were pressed into a square shape by using a pressing machine (model Eberhardt 2004, Lichtenau, Germany), then labeled and suspended on rods for further processing. The thermal processing consisting of hot smoking with pasteurization was conducted in a smoking chamber (UKM Junior 04, Mauting S.R.O., Valtice, Czech Republic) in three successive stages: an initial phase at 55 °C for 20 min, followed by 20 min at 65 °C, and a final steaming step at 80 °C until the product core reached 72 °C. After that, the sausages were cooled (0–4 °C). Beech wood shavings were used to produce smoke. For every formulation two production processes were carried out. The product, with a declared shelf life of 30 days, was stored under refrigerated conditions (0–4 °C) for the duration of the study.

### 4.3. Compositional Characteristics

The emulsion-type sausage samples were analyzed the day after processing for moisture, fat, and protein content following the AOAC official procedures [72]. Quantification of collagen was carried out following the procedure specified in the ISO 3496:1994 standard [73], applicable to meat and meat-derived products. Each analysis was performed in triplicate.

### 4.4. Color and pH Measurement

The color of the sausage samples was evaluated 24 h after production. Prior to analysis, the samples were homogenized with a high-speed blender to produce a homogeneous paste suitable for color determination. Color measurements were performed using a PCECSM1 Colorimeter (PCE Instruments, Southampton, UK), operating in the CIEL*a*b* color space based on spectral reflectance. Prior to analysis, the instrument was calibrated using the white reference standard supplied by the manufacturer. The color parameters L* (lightness), a* (+redness/−greenness), and b* (+yellowness/−blueness) were determined on three samples from each formulation, with measurements taken at five different locations on each sample. The total color difference (ΔE) was calculated according to the following equation:

$$∆E=\sqrt{{\left(L^{*}-L_{0}^{*}\right)}^{2}+{\left(a^{*}-a_{0}^{*}\right)}^{2}+{\left(b^{*}-b_{0}^{*}\right)}^{2}}$$

where ΔE is the total color difference between the reformulated sausage compared with control sausage; L_0_*, a_0_*, b_0_*—color coordinates of the control sausage; L*, a*, b*—color coordinates of the reformulated sausage.

The pH of the sausage samples was determined with a Hanna pH-meter (model HI255, Hanna Instruments, Padova, Italy). The pH values were measured in a slurry prepared at room temperature by high-speed blending of finely ground samples with distilled water in a 1:10 (*w*/*v*) ratio. The samples were analyzed in triplicate per formulation to calculate mean values.

### 4.5. Lipid Oxidation

The oxidative stability of the sausage was evaluated by measuring thiobarbituric acid-reactive substances (TBARS) one day after processing and after 30 days of refrigerated storage, by using the method of Witte et al. [74]. Briefly, sausage samples (5 g) were mixed using a vortex with 12.5 mL of 20% trichloroacetic acid, then transferred into a 25 mL volumetric flask and brought to volume with cold distilled water. After being centrifuged at 2500× *g* for 5 min, an aliquot of 5 mL from the supernatant was withdrawn and combined with an equal volume (5 mL) of 0.02 M 2-thiobarbituric acid. The resulting solution was subjected to heating at 100 °C for 35 min, then cooled to ambient temperature. Its absorbance was subsequently determined at 532 nm using a Varian Cary 50 UV spectrophotometer (Varian Co., Palo Alto, CA, USA). A standard curve was generated using 1,1,3,3-tetramethoxypropane, and the results were reported as mg of malondialdehyde (MDA) per kg.

### 4.6. Textural Properties

The textural properties of the samples were evaluated using a TVT-6700 texture analyzer (Perten Instruments, Hägersten, Stockholm, Sweden) by performing both compression and cutting tests. For the compression test, the texture analyzer was equipped with a 10 kg load cell, and all samples were prepared to a height of 15 mm; each sample underwent two consecutive compression cycles to 50% of its initial height using a cylindrical probe with a diameter of 20 mm. Before testing, the probe was positioned 5 mm above the sample surface. A trigger force of 20 g and a testing speed of 1.5 mm/s were used, with a 10 s pause between the two compression cycles to permit partial structural recovery. The parameters measured included hardness (N), adhesiveness (J), resilience, springiness, cohesiveness (all dimensionless), as well as gumminess (N) and chewiness (N), with each determination carried out in triplicate for every sample. For the cutting test, the texture analyzer was fitted with a high knife blade probe (117 mm) and a suitable probe holder. Measurements were carried out at the center of each sample. The probe was positioned 5 mm above the sample surface prior to testing. A test speed of 1.5 mm/s and a trigger force of 40 g were applied. This method enabled the determination of the force required to cut through the sample, expressed as shear force (N). Each measurement was performed in triplicate for every sample.

### 4.7. Sensory Analysis

The sensory evaluation of sausage samples was conducted in a university laboratory one day post-processing with a thirty-member panel comprising staff and master’s students from the Department of Food Science, University of Craiova (Craiova, Romania). Prior to participation, all panelists were informed about the principles and procedures of sensory evaluation. Written informed consent was obtained from each participant, confirming their voluntary agreement to take part in the study. A 9-point hedonic scale, ranging from 1 (“dislike extremely”) to 9 (“like extremely”), was used to evaluate appearance, taste, flavor, texture, and overall acceptability. Samples were presented in a randomized sequence using coded labels, while water and bread were provided for palate cleansing between tastings. All samples were assessed in triplicate, and mean values were calculated for each sensory attribute.

### 4.8. Statistical Analysis

Statistical analysis was performed using the Statgraphics Centurion XVI package (StatPoint Technologies, Warrenton, VA, USA). Results are expressed as mean ± standard deviation. The statistical significance of the effects of sausage formulation on compositional characteristics, color parameters, pH, and sensory properties was evaluated using one-way analysis of variance (ANOVA), followed by Fisher’s least significant difference (LSD) test at a 95% confidence level. In addition, two-way ANOVA, followed by the LSD test (*p* < 0.05), was used to assess the effect of storage time on TBARS values and textural properties of the sausage samples.

## Figures and Tables

**Table 1:** Compositional characteristics of control and reformulated sausage samples.

	CS	PC	PCC	PCCP	PCX	PCXP
Moisture (%)	59.88 ± 1.09 ^ab^	61.30 ± 0.89 ^a^	58.60 ± 0.92 ^bc^	58.87 ± 0.74 ^bc^	58.06 ± 0.91 ^c^	58.80 ± 1.24 ^c^
Protein (%)	16.52 ± 0.43 ^a^	17.20 ± 0.39 ^a^	17.18 ± 0.54 ^a^	16.89 ± 0.27 ^a^	16.89 ± 0.31 ^a^	17.13 ± 0.45 ^a^
Fat (%)	19.58 ± 0.64 ^c^	17.53 ± 0.48 ^d^	19.40 ± 0.33 ^c^	20.87 ± 0.41 ^b^	20.78 ± 0.56 ^b^	22.09 ± 0.61 ^a^
Collagen (%)	2.38 ± 0.09 ^b^	3.15 ± 0.13 ^a^	3.22 ± 0.12 ^a^	3.31 ± 0.15 ^a^	3.23 ± 0.08 ^a^	3.34 ± 0.10 ^a^
Collagen/Protein ratio (%)	14.41 ± 0.33 ^c^	18.31 ± 0.38 ^b^	18.74 ± 0.26 ^ab^	19.50 ± 0.49 ^a^	19.12 ± 0.43 ^a^	19.50 ± 0.53 ^a^

Different lowercase letters indicate significant differences between formulations (*p* < 0.05); CS—sausage made with soy gel and soy protein isolate (control); PC—sausage reformulated by replacing soy protein with collagen + plasma proteins; PCC—sausage reformulated by replacing soy protein with collagen + plasma proteins + 1% carrageenan; PCCP—sausage reformulated by replacing soy protein with collagen + plasma proteins + 1% carrageenan + 0.2% sodium tripolyphosphate; PCX—sausage reformulated by replacing soy protein with collagen + plasma proteins + 0.2% xanthan gum; PCXP—sausage reformulated by replacing soy protein with collagen + plasma proteins + 0.2% xanthan gum + 0.2% sodium tripolyphosphate.

**Table 2:** Color parameters (L*—lightness, a*—redness, b*—yellowness, C*—chroma and h*—hue angle) and pH of control and reformulated sausage samples.

	CS	PC	PCC	PCCP	PCX	PCXP
L*	51.53 ± 1.05 ^c^	55.69 ± 1.71 ^a^	54.29 ± 2.16 ^ab^	54.27 ± 1.53 ^ab^	52.84 ± 1.08 ^bc^	54.57 ± 1.22 ^ab^
a*	20.43 ± 0.87 ^ab^	20.84 ± 0.84 ^ab^	19.89 ± 1.06 ^b^	20.35 ± 0.80 ^ab^	19.84 ± 0.75 ^b^	20.25 ± 1.33 ^ab^
b*	14.05 ± 0.61 ^b^	15.27 ± 0.35 ^a^	14.07 ± 1.09 ^b^	15.38 ± 1.17 ^a^	14.38 ± 1.10 ^ab^	14.67 ± 0.61 ^ab^
C*	24.80 ± 1.01 ^abc^	25.83 ± 0.84 ^ab^	24.24 ± 1.26 ^c^	25.32 ± 1.32 ^ab^	24.68 ± 0.91 ^bc^	24.84 ± 1.72 ^abc^
h*	34.53 ± 0.73 ^b^	36.24 ± 0.80 ^a^	35.45 ± 1.15 ^ab^	35.73 ± 1.12 ^a^	35.36 ± 0.40 ^ab^	36.47 ± 0.80 ^a^
ΔE	-	4.35	2.81	3.05	2.47	3.10
pH	6.53 ± 0.08 ^a^	6.37 ± 0.01 ^c^	6.51 ± 0.03 ^ab^	6.53 ± 0.03 ^a^	6.45 ± 0.02 ^b^	6.46 ± 0.04 ^b^

Different lowercase letters indicate significant differences between formulations (*p* < 0.05); CS—sausage made with soy gel and soy protein isolate (control); PC—sausage reformulated by replacing soy protein with collagen + plasma proteins; PCC—sausage reformulated by replacing soy protein with collagen + plasma proteins + 1% carrageenan; PCCP—sausage reformulated by replacing soy protein with collagen + plasma proteins + 1% carrageenan + 0.2% sodium tripolyphosphate; PCX—sausage reformulated by replacing soy protein with collagen + plasma proteins + 0.2% xanthan gum; PCXP—sausage reformulated by replacing soy protein with collagen + plasma proteins + 0.2% xanthan gum + 0.2% sodium tripolyphosphate.

**Table 3:** Textural parameters of control and reformulated sausages at 0 and 30 days of storage at 4 °C.

Storage Time (Days)	Textural Parameters	CS	PC	PCC	PCCP	PCX	PCXP
Day 1	Hardness	49.25 ± 1.64 ^bB^	63.33 ± 3.50 ^aA^	63.59 ± 0.76 ^aB^	60.56 ± 2.08 ^aB^	30.56 ± 1.73 ^cA^	27.10 ± 0.64 ^cA^
	Adhesiveness	−16.77 ± 1.01 ^bB^	−11.08 ± 1.52 ^aB^	−24.55 ± 1.99 ^cB^	−19.50 ± 1.03 ^bB^	−26.97 ± 1.77 ^cB^	−17.66 ± 1.92 ^bB^
	Resilience	0.25 ± 0.01 ^cA^	0.30 ± 0.01 ^aA^	0.29 ± 0.00 ^aA^	0.27 ± 0.01 ^bA^	0.23 ± 0.01 ^dA^	0.22 ± 0.00 ^dA^
	Cohesiveness	0.53 ± 0.01 ^bcdB^	0.59 ± 0.02 ^abB^	0.65 ± 0.11 ^aA^	0.57 ± 0.03 ^abcA^	0.48 ± 0.02 ^dA^	0.49 ± 0.05 ^cdA^
	Gumminess	26.18 ± 0.74 ^cB^	36.94 ± 0.90 ^aB^	37.29 ± 1.53 ^aB^	34.06 ± 1.20 ^bB^	14.58 ± 1.02 ^dA^	13.19 ± 1.56 ^dA^
	Chewiness	31.44 ± 0.73 ^cB^	40.12 ± 2.05 ^abA^	41.98 ± 1.05 ^aB^	38.12 ± 1.48 ^bB^	17.45 ± 1.16 ^dA^	17.12 ± 0.81 ^dA^
	Shear force	26.86 ± 0.72 ^bB^	37.91 ± 0.80 ^aB^	40.37 ± 0.54 ^aB^	38.41 ± 1.28 ^aB^	22.11 ± 2.61 ^cB^	21.91 ± 1.70 ^cB^
Day 30	Hardness	59.26 ± 1.83 ^bA^	60.45 ± 1.54 ^bA^	66.95 ± 1.78 ^aA^	64.66 ± 0.62 ^aA^	29.90 ± 0.42 ^cA^	25.23 ± 0.76 ^dB^
	Adhesiveness	−13.71 ± 2.09 ^cA^	−2.47 ± 2.17 ^aA^	−3.74 ± 1.06 ^abA^	−6.15 ± 1.40 ^bA^	−21.79 ± 2.81 ^dA^	−12.84 ± 0.91 ^cA^
	Resilience	0.24 ± 0.01 ^cA^	0.29 ± 0.01 ^aA^	0.28 ± 0.01 ^aA^	0.26 ± 0.01 ^bA^	0.22 ± 0.01 ^dA^	0.21 ± 0.01 ^dA^
	Cohesiveness	0.64 ± 0.02 ^aA^	0.66 ± 0.01 ^aA^	0.69 ± 0.04 ^aA^	0.64 ± 0.06 ^aA^	0.51 ± 0.02 ^bA^	0.44 ± 0.04 ^cA^
	Gumminess	37.73 ± 2.35 ^cA^	40.38 ± 1.59 ^bcA^	45.36 ± 4.64 ^aA^	42.36 ± 1.74 ^abA^	15.28 ± 0.40 ^dA^	11.19 ± 1.16 ^dA^
	Chewiness	39.53 ± 1.07 ^cA^	42.13 ± 1.94 ^bcA^	47.28 ± 3.08 ^aA^	42.87 ± 0.89 ^bA^	18.77 ± 0.67 ^dA^	14.44 ± 0.75 ^eB^
	Shear force	41.50 ± 1.80 ^cA^	48.15 ± 1.66 ^bA^	59.39 ± 4.87 ^aA^	57.09 ± 7.16 ^aA^	26.99 ± 0.33 ^dA^	30.41 ± 0.21 ^dA^

Different lowercase letters in the same row indicate significant differences between sausage formulations (*p* < 0.05) for the same storage period, while different uppercase letters in the same column are indicative of significant differences between sampling times for the same sausage formulation (*p* < 0.05); CS—sausage made with soy gel and soy protein isolate (control); PC—sausage reformulated by replacing soy protein with collagen + plasma proteins; PCC—sausage reformulated by replacing soy protein with collagen + plasma proteins + 1% carrageenan; PCCP—sausage reformulated by replacing soy protein with collagen + plasma proteins + 1% carrageenan + 0.2% sodium tripolyphosphate; PCX—sausage reformulated by replacing soy protein with collagen + plasma proteins + 0.2% xanthan gum; PCXP—sausage reformulated by replacing soy protein with collagen + plasma proteins + 0.2% xanthan gum + 0.2% sodium tripolyphosphate.

**Table 4:** Sensory attributes and overall acceptability scores of control and reformulated sausage samples.

	CS	PC	PCC	PCCP	PCX	PCXP
Appearance	8.33 ± 0.72 ^a^	5.67 ± 1.18 ^c^	7.53 ± 0.74 ^b^	8.33 ± 0.62 ^a^	3.40 ± 0.74 ^d^	2.87 ± 0.64 ^d^
Color	8.20 ± 0.68 ^a^	6.60 ± 0.83 ^c^	7.40 ± 0.63 ^b^	8.33 ± 0.62 ^a^	4.13 ± 1.68 ^d^	4.13 ± 1.25 ^d^
Flavor	8.40 ± 0.63 ^a^	6.60 ± 1.12 ^b^	8.13 ± 0.64 ^a^	8.00 ± 0.76 ^a^	4.33 ± 0.82 ^c^	3.60 ± 0.63 ^d^
Taste	8.80 ± 0.41 ^a^	5.87 ± 1.06 ^c^	7.53 ± 0.74 ^b^	7.67 ± 0.98 ^b^	3.07 ± 0.59 ^d^	3.27 ± 0.88 ^d^
Texture	8.60 ± 0.51 ^a^	6.13 ± 0.74 ^c^	8.33 ± 0.62 ^ab^	7.80 ± 1.21 ^b^	2.87 ± 0.83 ^d^	3.13 ± 0.99 ^d^
General acceptability	8.53 ± 0.52 ^a^	6.33 ± 0.62 ^c^	8.00 ± 0.65 ^b^	8.27 ± 0.80 ^ab^	3.73 ± 0.80 ^d^	3.87 ± 0.74 ^d^

Different lowercase letters indicate significant differences between formulations (*p* < 0.05); CS—sausage made with soy gel and soy protein isolate (control); PC—sausage reformulated by replacing soy protein with collagen + plasma proteins; PCC—sausage reformulated by replacing soy protein with collagen + plasma proteins + 1% carrageenan; PCCP—sausage reformulated by replacing soy protein with collagen + plasma proteins + 1% carrageenan + 0.2% sodium tripolyphosphate; PCX—sausage reformulated by replacing soy protein with collagen + plasma proteins + 0.2% xanthan gum; PCXP—sausage reformulated by replacing soy protein with collagen + plasma proteins + 0.2% xanthan gum + 0.2% sodium tripolyphosphate.

**Table 5:** Formulations (kg) of the different emulsion-type sausage samples.

	CS	PC	PCC	PCCP	PCX	PCXP
Pork trims (80/20)	11.00	11.00	11.00	11.00	11.00	11.00
Pork leg meat	3.43	3.43	3.43	3.43	3.43	3.43
Pork backfat	2.74	2.74	2.74	2.74	2.74	2.74
Water	2.71	5.28	5.05	5.00	5.23	5.18
Soy protein gel	3.43	-	-	-	-	-
Soy protein isolate	0.35	-	-	-	-	-
Combi Gel P	-	0.43	0.43	0.43	0.43	0.43
Combi Gel PP Collagen	-	0.78	0.78	0.78	0.78	0.78
Carrageenan	-	-	0.23	0.23	-	-
Xanthan	-	-	-	-	0.05	0.05
Sodium tripolyphosphate	-	-	-	0.05	-	0.05
Additives and spices mixture	0.62	0.62	0.62	0.62	0.62	0.62
Nitrite curing salt	0.37	0.37	0.37	0.37	0.37	0.37
Sodium lactate	0.35	0.35	0.35	0.35	0.35	0.35
Total	25	25	25	25	25	25

## Data Availability

The original contributions presented in this study are included in the article. Further inquiries can be directed to the corresponding author.

## Abbreviations

The following abbreviations are used in this manuscript:

MDA	Malondialdehyde
ΔE	Total color difference
TBARS	Thiobarbituric acid-reactive substances
